# Functional brain growth trajectories across the first decade of life from a single longitudinal cohort

**DOI:** 10.21203/rs.3.rs-9557891/v1

**Published:** 2026-05-14

**Authors:** Haitao Chen, Janelle Liu, Yifan Gao, Kaiqiao Han, Joe Blocher, Emil Cornea, Yizhou Sun, Debiao Li, John H. Gilmore, Wei Gao

**Affiliations:** 1 Cedars-Sinai Health Sciences University Biomedical Imaging Research Institute, Cedars-Sinai Medical Center, Los Angeles, CA; 2 Department of Biomedical Sciences, Cedars-Sinai Medical Center, Los Angeles, CA; 3 Department of Bioengineering, University of California, Los Angeles, Los Angeles, CA; 4 Department of Computational Biomedicine, Cedars-Sinai Medical Center, Los Angeles, CA; 5 Tsinghua Medicine, Tsinghua University, Beijing, China; 6 Department of Computer Science, University of California, Los Angeles, Los Angeles, CA; 7 Department of Psychiatry, University of North Carolina Chapel Hill, Chapel Hill, NC

## Abstract

The first decade of life is marked by rapid reorganization of functional brain networks, yet normative longitudinal trajectories of key properties characterizing brain functional connectivity remain poorly defined. Using 1,436 resting-state fMRI scans from 633 children followed from birth to 10 years, we derived the first normative growth trajectories of functional connectivity maturation, network topology, and large-scale functional gradients. Network development followed a hierarchical, nonlinear pattern from primary to transmodal systems. Children later diagnosed with neurodevelopmental disorders showed disrupted functional gradient trajectories. Early measures of functional connectivity predicted later socioemotional and cognitive outcomes at 8 and 10 years of age. A deep learning brain-age prediction model revealed disorder-specific deviations, distinguishing risk groups in early childhood. Together, these findings establish normative reference curves for functional brain development and demonstrate the prognostic value of early functional connectivity measures, offering novel imaging biomarkers for the early identification of neurodevelopmental risk.

The first decade of life is a critical period for human brain development when dramatic structural and functional changes drive the formation of foundational neural circuits that support cognitive, socioemotional, and behavioral processes across the lifespan.^1–3^ The developing functional connectome is shaped by genetic and environmental factors,^4–6^ including maternal mental health^7–12^ and risk for neurodevelopmental disorders,^13–20^ which influence long-term developmental outcomes.^1,21–24^ Most existing work on brain development has focused on discrete developmental periods in early infancy^25^ or later childhood.^26^ Moreover, efforts to understand longitudinal growth of the functional connectome have largely relied on cross-sectional developmental modeling,^27^ highlighting a critical need to map continuous growth trajectories of the brain’s functional organization across the first decade of life. Further, understanding how these developmental trajectories are impacted by maternal mental health and neurodevelopmental disorders is essential for early risk stratification and the development of timely interventions.

Recent work has begun to delineate whole-brain and network-level growth curves charting changes in brain functional organization across time, but truly longitudinal functional connectivity growth studies spanning the first decade of life remain sparse. A recent lifespan study examining changes from birth to 80 years of age identified critical inflection points in whole-brain connectome properties throughout the third and fourth decades of life,^28^ but broad lifespan models lack the granularity needed to capture rapid neurodevelopmental shifts in early childhood. Developmental studies examining growth across the first 6 years of life have demonstrated dramatic growth with different functional brain networks maturing at different rates,^26,27,29^ but little is known about continuous neurodevelopment through 10 years of age. Thus, although these large-scale studies have greatly improved our understanding of normative growth curves of brain functional organization, several critical gaps remain. First, these studies tend to aggregate data across multiple cross-sectional datasets to generate longitudinal growth curves. This significantly enhances statistical power but introduces blurring effects due to high individual variability in functional connectivity^30^ and prevents accurate brain-based prediction of later behavioral outcomes.^31^ Second, even when longitudinal datasets are included (e.g., Yin et al., 2025), these efforts often aggregate multiple smaller cohorts with limited within-cohort age coverage (i.e., no single dataset spans the full age range examined). This may introduce cohort-related effects that complicate the interpretation of the generated developmental trajectories. Third, despite substantial evidence that neurodevelopmental risk factors alter early growth trajectories across the first 2 years,^7,9,13,32–36^ it is still unknown how these factors shape longitudinal functional connectivity growth trajectories from infancy through late childhood. A thorough understanding of how brain trajectories across the first decade are shaped by risk factors, including neurodevelopmental disorders and maternal mental health, would enable more robust and earlier neuroimaging-based prediction of later developmental outcomes.

Here, we leveraged data from a longitudinal single-cohort study including 1,436 resting-state functional magnetic resonance imaging (rsfMRI) scans acquired from 633 unique individuals with scans at 3 weeks (neonate) and 1, 2, 4, 6, 8, and 10 years of age (Table S1). Behavioral assessments were collected at 8 and 10 years of age, with confirmed clinical diagnosis of any neurodevelopmental disorder at 4 years of age. We aimed to: 1) Create the first set of longitudinal growth curves charting development of the brain’s functional organization across the first decade using a subset of full-term typically developing controls (TD; n=379); 2) Assess risk-related alterations in a subset of participants with a confirmed neurodevelopmental disorder diagnosis (NDD subgroup; diagnosis with attention-deficit/hyperactivity disorder [ADHD] or autism; n=36) and another subset of participants whose mothers had a psychiatric diagnosis (MPD subgroup; maternal diagnosis with schizophrenia, schizoaffective, bipolar, major depression, psychosis, and mood disorders; n=100); 3) Test whether early neuroimaging parameters predict 8- and 10-year behavioral outcomes assessing intelligence (intelligence quotient; IQ), working memory, anxiety, and depression; and 4) Apply deep learning-based brain age prediction and evaluate brain age gap as a functional connectivity marker of neurodevelopmental risk.

## Results

### Normative growth curves of key brain functional connectivity properties

To characterize functional brain development across the first ten years of life, we examined three key functional connectivity properties: 1) A maturation score (Mscore), defined as the cosine similarity between each voxel’s whole-brain functional connectivity pattern with that of a corresponding adult reference; 2) Graph theory measures,^37^ including nodal strength, nodal global efficiency, and nodal local efficiency, that quantify the ease of information exchange within the global brain system; and 3) Functional gradients,^38^ which quantify large-scale functional organization by mapping brain regions along continuous axes of whole-brain connectivity similarity; these have been shown to index critical developmental changes across the lifespan.^39^ Briefly, each functional connectivity parameter was first calculated at the voxel level to generate qualitative heatmaps. Average network- and region of interest (ROI)-level measures were then derived to identify cross-sectional patterns of growth in each age group (i.e., at 3 weeks and 1, 2, 4, 6, 8, and 10 years) as well as longitudinal growth trajectories across all age groups (using multivariate sparse functional principal components analysis [mSFPCA]^40^; see Supplementary Information for details).

**Figure 1.**
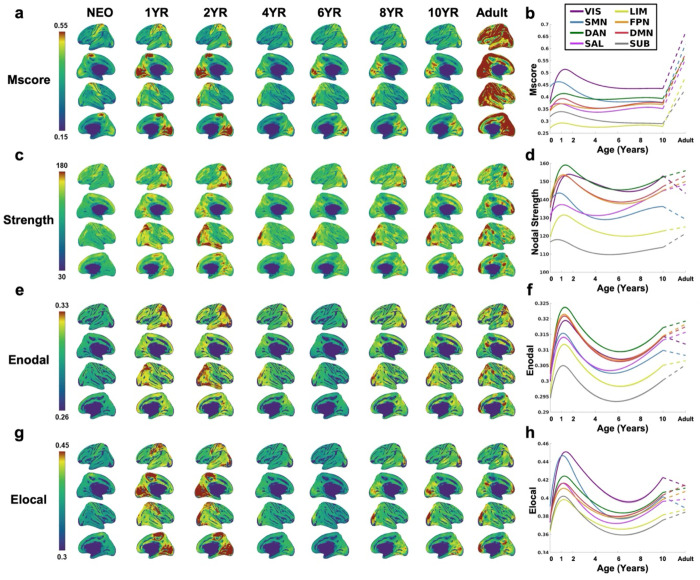
Normative heatmaps and growth trajectories of functional maturation and network topology from birth to 10 years. Maturation scores (Mscore) increased overall with age (**a**), but followed network-specific sequences (**b**). Graph theory measures showed a similar nonlinear pattern for nodal strength (Strength, **c-d**), nodal global efficiency (Enodal, **e-f**), and nodal local efficiency (Elocal, **g-h**). Network plots (b, d, f, h) are shown with standard error of the mean. [DAN: dorsal attention network, DMN: default mode network, FPN: frontoparietal network, LIM: limbic network, SAL: salience network, SMN: sensorimotor network, SUB: subcortical network, VIS: visual network]

Maturation scores increased with age (Figure 1a), with different networks maturing at different rates (Figure 1b). At birth, the sensorimotor network showed the highest maturation score (i.e., across all networks, the sensorimotor network had the highest level of adult-like topology at birth). By 1 year, the visual network showed the highest maturation score. Across all networks, maturation scores decreased from 2 to 4 years of age, followed by a relative plateauing from 4 to 10 years of age. Extrapolating from age 10 to adulthood, our results suggest that maturation scores likely continue to increase across adolescence in all networks to reach adult levels.

All graph theory measures, including nodal strength (Figure 1c–d), nodal global efficiency (Figure 1e–f), and nodal local efficiency (Figure 1g–h), showed similar developmental patterns with initial increases peaking at or before 2 years of age, followed by regression with scores bottoming out at 6 years, then increasing through 10 years of age. Extrapolating from 10 years to adulthood, most networks showed higher graph theory measure scores in adulthood (i.e., stronger information exchange efficiency in adults), suggesting that increases in functional network efficiency from 6 to 10 years continue through adulthood. However, primary visual and sensorimotor networks were the only networks with higher graph theory measure scores at age 10 relative to adulthood, suggesting that visual and sensorimotor systems may exhibit earlier developmental peaks in information exchange efficiency.

**Figure 2.**
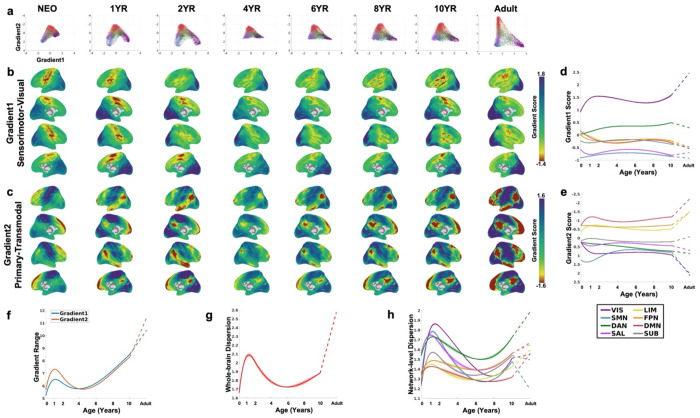
Normative heatmaps and developmental trajectories of functional gradients from birth to 10 years. Group-level gradient maps illustrate the emergence of large-scale functional organization across age along two principal axes (**a**): Gradient1 anchored by sensorimotor and visual systems (sensorimotor-visual axis, **b**) and Gradient2 spanning primary to transmodal regions (primary-transmodal axis, **c**). Longitudinal growth trajectories show early maturation of Gradient1 (**d**) and protracted development of Gradient2 (**e**), with the dominant organizational axis shifting from Gradient1 to Gradient2 at 2 years of age (**f**). Gradient dispersion scores peaked at 1 year and declined through 5 years before increasing again through 10 years at both the whole-brain (**g**) and network (**h**) levels, reflecting nonlinear changes in functional segregation across development. [DAN: dorsal attention network, DMN: default mode network, FPN: frontoparietal network, LIM: limbic network, SAL: salience network, SMN: sensorimotor network, SUB: subcortical network, VIS: visual network]

Consistent with the developmental patterns observed for functional maturation scores and graph theory measures, functional gradients matured most rapidly during the first two years, with fewer fluctuations in functional gradient scores from 2 to 10 years of age. At birth, a primitive version of adult functional connectivity gradient patterns was observed (Figure 2a), with one gradient anchored by sensorimotor and visual areas (Gradient1, sensorimotor-to-visual gradient, Figure 2b) and the other anchored by primary and transmodal regions (Gradient2, primary-to-transmodal gradient, Figure 2c). Following early maturation from birth to 2 years, Gradient1 scores stabilized through 10 years of age for all networks. The only exception to this pattern was the visual network, which showed an inflection in Gradient1 score (i.e., increased separation of brain regions along this gradient) starting at 10 years of age towards adult-like levels (Figure 2d). By age 10, Gradient2 scores for the three transmodal networks (default mode, frontoparietal, and limbic networks) had not yet reached adult values, whereas scores for the two primary networks (visual and sensorimotor networks) exceeded their adult values (Figure 2e). This suggests that polarization along Gradient2 likely occurs after 10 years of age, pointing to adolescence as an important developmental period when socioemotional and executive control networks dominate the functional hierarchy. Indeed, there was a shift from Gradient1 (sensorimotor-to-visual) to Gradient2 (primary-to-transmodal) as the dominant gradient at 2 years of age (Figure 2f). Functional gradient dispersion scores, which reflect gradient segregation at whole-brain level,^41^ peaked at 1 year, then decreased through 5 years before increasing again through 10 years of age (Figure 2g). Similar nonlinear trajectories were observed for network-level functional gradient dispersion scores (Figure 2h).

### Altered brain development associated with neurodevelopmental risk factors

**Figure 3.**
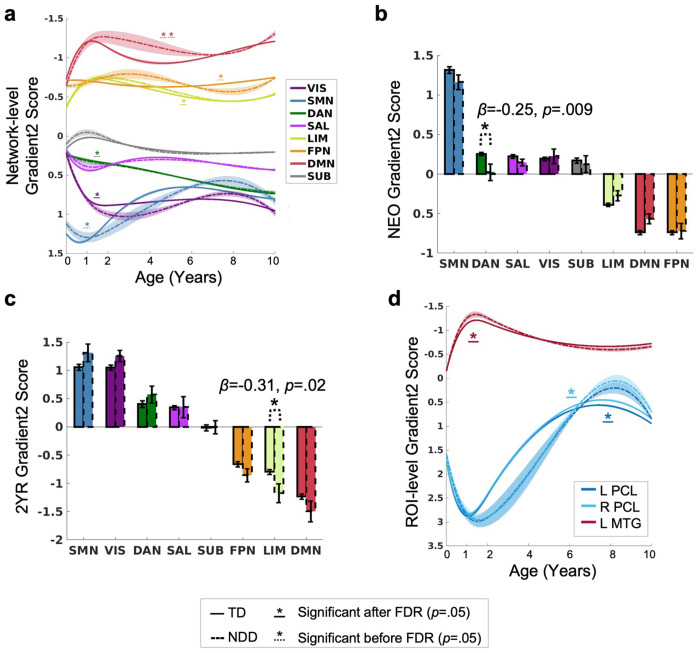
Altered functional gradient development in children with neurodevelopmental disorders. Group differences in longitudinal growth trajectories of the primary-to-transmodal gradient (Gradient2) were observed between typically developing controls (TD, solid lines) and children later diagnosed with neurodevelopmental disorders (NDD, dashed lines), particularly in the visual and limbic networks (**a**). Post-hoc cross-sectional analyses revealed that compared to TD, NDD had lower dorsal attention network Gradient2 scores at birth (**b**) and lower limbic network Gradient2 scores at 2 years of age (**c**). ROI-level analysis identified group-specific differences on growth trajectories of the left and right paracentral lobules (PCL) and left middle temporal gyrus (MTG) (**d**). [DAN: dorsal attention network, DMN: default mode network, FPN: frontoparietal network, LIM: limbic network, SAL: salience network, SMN: sensorimotor network, SUB: subcortical network, VIS: visual network]

We next sought to examine how neurodevelopmental risk factors associated with neurodevelopmental disorder diagnosis and maternal psychiatric diagnosis may impact functional brain growth trajectories. Using a subgroup of participants with a confirmed neurodevelopmental disorder diagnosis (NDD) and a subgroup of participants whose mothers had a confirmed psychiatric diagnosis (MPD), we characterized longitudinal growth trajectories for all functional connectivity properties and evaluated whether they deviated from that observed in neurotypical controls. Briefly, for each functional connectivity parameter, each individual’s growth trajectory was modeled as the weighted sum of the group mean curve and the first two functional principal component (FPC) curves generated from mSFPCA (see Supplementary Information), and the corresponding FPC scores (i.e., weights of the two mSFPCA FPCs) were used as longitudinal features. Across all functional connectivity properties, group differences were observed only in the development of functional connectivity gradients. Specifically, NDD showed altered Gradient2 growth trajectories, with significant effects before false discovery rate (FDR) correction in the sensorimotor (FPC1: *p*=.03), visual (FPC2: *p*<.01), dorsal attention (FPC1: *p*=.04), limbic (FPC1: *p*<.01), frontoparietal (FPC1: *p*=.04), and default mode networks (FPC1: *p*=.02; FPC2: *p*=.02) (Figure 3a). Among these, NDD effects on visual and limbic networks remained significant after FDR correction (Figure S1a). To home in on the age at which these trajectory differences emerge, we conducted post-hoc cross-sectional analyses to assess group differences within each age group. Compared to TD, NDD had lower dorsal attention network Gradient2 scores at birth (*p*<.01; Figure 3b) and lower limbic network Gradient2 scores at 2 years (*p*=.02; Figure 3c). Next, to capture regional growth patterns, we examined NDD effects on ROI-level growth trajectories (Figure 3d). Compared to TD, NDD showed significant differences in Gradient2 growth trajectories after FDR correction in the left paracentral lobule (PCL, *p*<.001), right PCL (*p*<.001) and left middle temporal gyrus (MTG, *p*<.001) (Figure S1b). We did not observe any effects of MPD on developmental trajectories of any functional connectivity properties (i.e., no significant differences between TD and MPD).

### Post-hoc analyses confirm reproducibility of global and network growth curves

**Figure 4.**
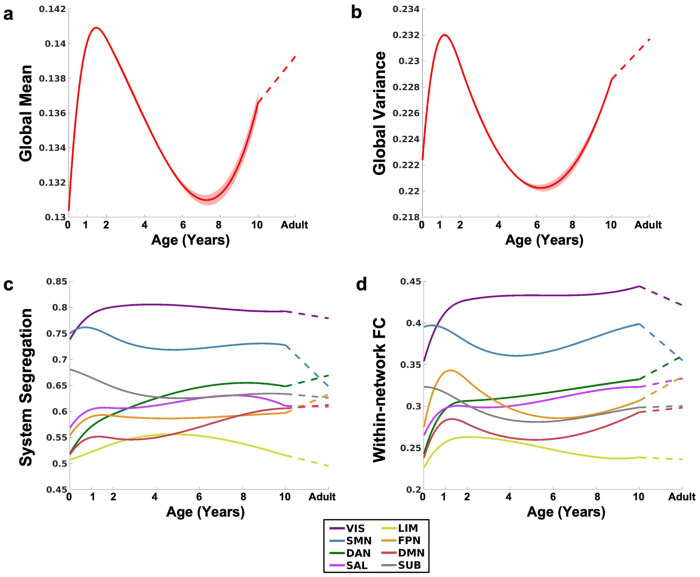
Robustness analysis comparing developmental trajectories with previously reported multisite datasets confirm reproducibility of global and network-level growth curves. Across both global (**a-b**) and network-level (**c-d**) measures, growth curves derived from the present longitudinal cohort closely match those reported in prior multisite studies (Sun et al., 2025: **a, c**; Yin et al., 2025: **b, d**), demonstrating the consistency of developmental trends across datasets. [DAN: dorsal attention network, DMN: default mode network, FPN: frontoparietal network, LIM: limbic network, SAL: salience network, SMN: sensorimotor network, SUB: subcortical network, VIS: visual network]

In a post-hoc robustness analysis, we tested whether the growth curves from 0 to 10 years estimated based on our single longitudinal cohort were consistent with those derived from aggregated multi-site datasets. We first reconstructed the growth curves of several global and network-level functional connectivity parameters reported in two previous studies.^28,29^ Briefly, global parameters included the mean and variance of voxel-level functional connectivity values,^28^ and network-level parameters included measures of system segregation^28^ and within-network functional connectivity.^29^ Global (Figure 4a–b) and network-level parameters (Figure 4c–d) were largely consistent with those reported in Sun et al. and Yin et al., respectively. We did not observe alterations associated with neurodevelopmental risk factors (i.e., NDD or MPD), suggesting that these global and network-level parameters represent general developmental trends but may lack the sensitivity to detect subtle changes associated with the examined risk factors.

### Early functional connectivity predicts later socioemotional and cognitive outcomes

Across all brain parameters examined, significant changes were observed from birth through 10 years, but the most dramatic development consistently occurred across the first 2 years. We therefore examined whether early brain connectivity at 3 weeks, 1 year, and 2 years predicted behavioral outcomes at 8 and 10 years of age. Briefly, we tested whether canonical correlation analysis (CCA) prediction models trained on neurotypical infants (n=379) could generalize to an independent test dataset spanning a range of neurodevelopmental outcomes (n=254). Functional connectivity features for each age group included ROI-level brain features to optimally balance between dimensionality and granularity to enable robust cross-cohort prediction. Prediction targets were 8- and 10-year anxiety, depression, and working memory scores (see Supplementary Information).

**Figure 5.**
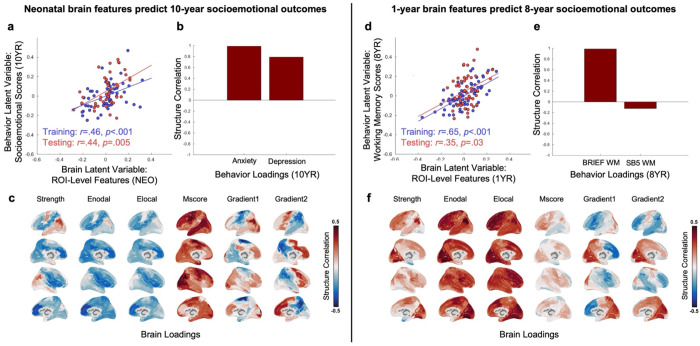
Early functional connectivity predicts later behavioral outcomes. Neonatal functional connectivity features predicted 10-year socioemotional outcomes, including anxiety and depression as indexed by the BASC-2 (**a-b**). Across the six brain features, higher neonatal maturation scores reflecting earlier functional maturation were associated with higher 10-year anxiety and depression scores (**c**). 1-year functional connectivity features were associated with 8-year cognitive outcomes, including working memory as indexed by the BRIEF and SB-5 (**d-e**). Across the six brain features, higher nodal global efficiency and nodal local efficiency at 1 year predicted poorer 8-year working memory performance (**f**). [BASC-2: Behavior Assessment System for Children, 2^nd^ edition; BRIEF: Behavior Rating Inventory of Executive Function; Elocal: nodal local efficiency; Enodal: nodal global efficiency; Gradient1: sensorimotor-to-visual gradient; Gradient2: primary-to-transmodal gradient; Mscore: functional connectivity maturation score; SB-5: Stanford-Binet Intelligence Scales, 5^th^ edition; Strength: nodal strength]

Two significant associations were observed. First, a set of neonatal functional connectivity features (including neonatal maturation score, nodal global efficiency, nodal local efficiency, nodal strength, Gradient1 score, and Gradient2 score) predicted 10-year socioemotional outcomes (anxiety and depression) (Figure 5a–b). Specifically, earlier maturation (i.e., higher maturation scores at 3 weeks) was associated with higher anxiety and depression scores at 10 years of age (Figure 5c). Second, a set of 1-year functional connectivity features predicted 8-year working memory outcomes (Figure 5d–e), with higher nodal global efficiency and nodal local efficiency at 1 year predicting worse working memory capabilities at 8 years of age (Figure 5f).

### Evaluating brain age gap as a biomarker for neurodevelopmental risk and diagnosis

**Figure 6.**
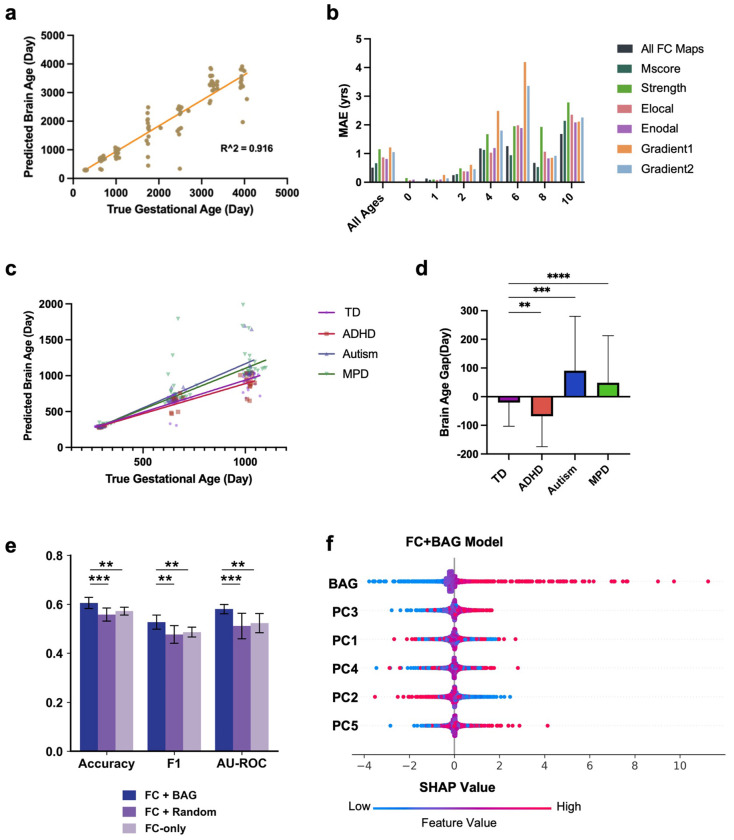
Functional connectivity-derived brain age reveals distinct developmental deviations in neurodevelopmental risk groups. The brain age regression model trained on TD participants achieved high predictive accuracy (MAE=0.51 years, RMSE=1.03 years, *R*^2^=0.92) (**a**). Prediction accuracy was highest when including all functional connectivity (FC) features/maps compared with models that included only one functional connectivity feature at a time (**b**). Across age groups, prediction accuracy was highest at 0, 1, and 2 years of age (i.e., lowest MAE values at 0, 1, and 2; **b**). This model was applied to the risk cohorts, including participants with a neurodevelopmental disorder diagnosis (ADHD or autism) and participants whose mothers had a psychiatric diagnosis (MPD) (**c**). Significant shifts in brain age gap (BAG) in ADHD, autism, and MPD were observed relative to TD, with ADHD showing negative mean BAG, reflecting maturational delay, and autism and MPD showing positive mean BAG, reflecting maturational acceleration (**d**). BAG improved classification performance beyond functional connectivity features alone in distinguishing TD participants from neurodevelopmental risk groups (NDD and MPD): the FC+BAG model outperformed the FC-only and FC+Random models across all metrics (**e**), with BAG ranking as the most influential feature (**f**). [BAG: brain age gap; Elocal: nodal local efficiency; Enodal: nodal global efficiency; Gradient1: sensorimotor-to-visual gradient; Gradient2: primary-to-transmodal gradient; Mscore: functional connectivity maturation score; PC: functional connectivity principal component; Strength: nodal strength]

Lastly, we trained a deep learning-based brain age prediction model to determine if early functional connectivity measures could classify risk groups (MPD) and predict clinical diagnosis (NDD). All six functional connectivity parameters were included in this model, which was trained on a subsample of TD participants (n=379). The model achieved a high regression performance with mean absolute error (MAE)=0.51 years, root mean squared error (RMSE)=1.03 years, and *R^2^*=0.92 (Figure 6a) on held-out TD participants. Consistency was highest using ensemble parameters between birth and 2 years of age (Figure 6b), suggesting that functional connectivity-derived features are especially predictive during early development. When we applied this model to the neurodevelopmental risk cohorts (MPD and NDD; Figure 6c), there were significant brain age gap (BAG) shifts across 3 weeks, 1 year, and 2 years relative to the TD group (Figure 6d). Since ADHD and autism are known to be associated with different brain connectivity patterns,^42,43^ we examined these clinical groups separately. Further analysis revealed distinct neurodevelopmental profiles, with ADHD subgroup showing a negative mean BAG, reflecting maturational delay (i.e., lower brain age relative to chronological age), and the autism and MPD subgroups showing a positive mean BAG, reflecting accelerated development (i.e., higher brain age relative to chronological age). This suggests that BAG is not only a marker of general risk but may also be able to distinguish between specific clinical diagnoses, including functional signatures of ADHD and autism. Finally, to assess the diagnostic power of BAG, we tested whether whole-brain BAG improved classification performance beyond functional connectivity features alone in distinguishing TD participants from neurodevelopmental risk groups (NDD and MPD). We compared three models: 1) an FC+BAG model, which included five functional connectivity features (reduced via principal components analysis from six functional connectivity features across eight networks to five principal components) plus BAG; 2) an FC-only model; and 3) an FC+Random model, which included functional connectivity features and a random Gaussian feature matched to the mean and standard deviation of BAG (see Methods). The FC+BAG model significantly outperformed both comparison models across all metrics. Relative to the FC-only model, the FC+BAG model improved accuracy by 3.3% (*p*<.001), F1 score by 3.1% (*p*<.01), and AUROC by 5.7% (*p*<.001). Compared to the FC+Random model, it improved accuracy by 4.7% (*p*<.01), F1 score by 5.0% (*p*<.01), and AUROC by 6.9% (*p*<.01; Figure 6e, Table 1). Across all top-performing models, BAG was the most influential feature (mean |SHAP|=.40; Figure 6f). By contrast, the matched random feature ranked substantially lower in importance (mean |SHAP|=.22; Figure S2), indicating that BAG contributes meaningful diagnostic information rather than reflecting a dimensionality artifact.

## Discussion

In this study, we derived the first set of normative growth curves characterizing six fundamental functional connectivity properties of network development across the first decade of life, including functional connectivity maturation, graph-theoretical organization, and large-scale functional gradients. Deviations in the developmental trajectories of functional gradients were observed in a subgroup of children diagnosed with a neurodevelopmental disorder (ADHD or autism), suggesting that alterations in macroscale cortical organization emerge early in life with cascading effects across the first decade. Brain–behavior analyses demonstrated the long-term predictive value of early functional connectivity. Neonatal maturation scores significantly predicted anxiety and depression scores at 10 years, and 1-year graph theory measures predicted working memory at 8 years. These findings indicate that early functional network architecture meaningfully predicts later socioemotional and cognitive outcomes. Finally, our deep learning–based brain age analysis revealed distinct patterns of developmental deviation across clinical groups. Relative to TD trajectories, children with ADHD showed delayed functional maturation, whereas children with autism and MPD showed accelerated maturation. Together, these results establish robust normative reference curves for functional connectivity development and highlight the potential of early-life functional connectivity measures (ages 0-2) to differentiate neurotypical development from high-risk trajectories and to predict behavioral outcomes in late childhood.

A hierarchical sequence of network maturation was observed, progressing from primary to higher-order systems and underscoring the foundational role of primary networks in early brain development.^44^ Our findings are highly consistent with previous work showing that lower-order primary networks develop first, with higher-order networks experiencing protracted development.^44^ Indeed, the sensorimotor network matured the earliest (i.e., highest maturation scores at birth), and the visual network rapidly matured postnatally to have the highest maturation scores by ~1 year, consistent with developmental milestones where visual experience becomes increasing salient for infants.^44^ Higher order networks, including the limbic, subcortical, salience, frontoparietal, and default mode networks, were slower to mature, with lower maturation scores across the first decade.

This general pattern of development was also reflected by the graph theory measures we examined. Primary systems, including sensorimotor and superior frontal regions, showed the highest nodal strength in neonates, consistent with prior studies^45–51^ identifying these areas as early functional hubs^52–58^ supporting perception-action processes. During toddlerhood, dorsal attention network regions were the next to mature, as indexed by high nodal strength and nodal global efficiency, reflecting their role as a transient integrative hub linking primary sensory systems with emerging higher-order networks during this period of rapid connectome reorganization.^44,52^ These regions support visuospatial attention, visuomotor integration, and top-down cognitive control,^53–56^ and their prominence during this period may reflect the integration of rapidly developing sensory and attentional processes.^33,57–59^ Consistent with prior work,^58^ network organization also shifted during the first year from predominantly provincial hubs (within-network connectivity) in sensorimotor areas toward increasing connector hubs (between-network connectivity) between heteromodal association areas. Similarly, graph-theory efficiency metrics showed a nonlinear trajectory across development. Nodal global efficiency increased sharply across the first year, likely reflecting downstream functional connectivity increases due to rapid synaptogenesis and axonal growth.^60^ In line with previous work,^47,61,62^ nodal global efficiency remained elevated through ~2 years before decreasing gradually through middle childhood, with another gradual increase starting ~10 years of age.^54,77,78^ This nonlinear pattern of growth may reflect synaptic pruning and the evolving balance between network integration and segregation.^63^ Nodal local efficiency showed a similar increase across the first year, followed by a decline from through 8 years before a subsequent increase towards 10 years of age, consistent with a transition from short- to long-range connectivity in toddlerhood.^26,47^ Overall, higher-order networks showed greater nodal global efficiency, whereas primary networks showed higher nodal local efficiency, consistent with their roles in distributed transmodal integration versus localized unimodal processing.

Functional gradient analyses revealed three key developmental patterns. First, the most pronounced nonlinear changes in functional gradient development occurred during the first four years, highlighting this period as a critical window for large-scale network reorganization.^1,25–27,64^ In line with previous studies,^65,66^ neonates showed a dominant sensorimotor-to-visual gradient and a relatively immature primary-to-transmodal gradient. Both gradients evolved rapidly during the first two years, with increasing differentiation between sensorimotor versus visual networks along the first gradient and between primary versus transmodal networks along the second gradient. Gradients continued to refine through age 4, resulting in adult-like topologies by 4 years, with gradual specialization through later childhood.^67–69^

Second, we observed developmental shifts in the relative dominance of the two gradients. At birth, the gradients showed comparable gradients, with sensorimotor-to-visual organization slightly dominant, in line with previous work.^66^ During infancy and early childhood (0-4 years), the primary-to-transmodal gradient had a larger range of values than that observed for the sensorimotor-to-visual gradient; this was driven by the specialization of primary networks along this axis coupled with the emergence of transmodal networks, including the default mode network.^70^ By 4 years of age, gradient topology stabilized and the sensorimotor-to-visual gradient showed a larger range through late childhood (4-10 years), before a later transition to the primary-to-transmodal gradient showing a larger range shortly after 10 years of age, marking the beginning of the transition from childhood to adolescence.^68,69^ These shifts in gradient range highlight the nonlinear emergence of large-scale functional hierarchy.

Third, whole-brain and network-level gradient dispersion captured evolving patterns of functional connectivity integration and segregation. During early development, the equilibrium between integration and segregation is vital for efficient information processing and the maturation of cognitive functions.^63,71,72^ Integration (increasing strength of long-range connectivity) facilitates communication and coordination across various brain regions, whereas segregation (increasing strength of short-range connectivity) supports the specialization of these regions for distinct processes. Consistent with other reports,^63,68,73,74^ whole-brain gradient dispersion increased during the first year, consistent with rapid proliferation of local connectivity, then declined through early childhood before increasing again toward, coinciding with synaptic pruning and myelination^60,75,76^ that contributes to refinement and segregation of primary and transmodal networks. At the network level, higher-order networks (default mode, frontoparietal control, and attention networks) showed gradual, protracted changes in dispersion scores, consistent with previous reports of extended development in these networks.^44^ By contrast, primary networks (visual, sensorimotor) showed more dynamic changes in dispersion score, with dramatic increases in dispersion over the first year, reflecting specialization following established synchronization at birth,^44,64^ followed by decreases from ages 2 to 6, reflecting functional integration as the functional connectome matures. Indeed, the observed dispersion pattern aligns with the previously-established functional connectivity maturation sequence from lower- to higher-order networks,^44^ extending this to reflect the complex interplay of network-level synchronization and specialization across infancy and childhood.

Altered longitudinal trajectories of functional connectivity were detected in children later diagnosed with neurodevelopmental disorders (ADHD or autism). At the network level, these children showed delayed maturation of the primary-to-transmodal gradient, particularly in sensorimotor, visual, limbic, and default mode networks. Cross-sectional analyses further revealed gradient alterations in dorsal attention and limbic networks in NDD neonates and 2-year-olds, indicating that disrupted large-scale network organization associated with risk for neurodevelopmental disorders emerges early in infancy. ROI-level findings were consistent, with bilateral PCL and left MTG showing altered primary-to-transmodal gradient score growth trajectories. These results align with previous reports of altered functional connectivity in ADHD^42,77–79^ and autism^80–89^ that emerge in early infancy^13,14,32,34,90^, most consistently involving connectivity in the default mode, dorsal attention, limbic, sensorimotor, and cognitive control networks.^45,93–95^ Furthermore, studies examining disorder-associated alterations in functional gradients have identified atypical hierarchical organization between sensory and transmodal regions in adolescents and young adults with autism.^43^ Our results extend this literature by demonstrating that aberrant primary-to-transmodal specialization can be detected during the first years of life and tracked longitudinally across the first decade.

Early functional connectivity also showed meaningful associations with later socioemotional and cognitive outcomes. Accelerated neonatal maturation scores predicted greater anxiety and depression symptoms at 10 years, indicating that deviations in the pace of network development, rather than simply delayed endpoints, may confer risk. Indeed, our findings suggest that accelerated maturation may represent a deviation from the typical developmental program, potentially establishing neural architecture that is less adaptive and more vulnerable to psychopathology.^91,92^ The neonatal period is characterized by rapid yet precisely-timed synaptogenesis and network formation.^93^ Overly rapid establishment of functional connections could lead to a system that is prematurely rigid, which could impair the brain’s ability to flexibly adapt to environmental inputs and stressors throughout childhood, a key factor in developing emotional resilience.^94^ Furthermore, circuits involving the amygdala, prefrontal cortex, and other default mode network regions, which are central to emotion regulation, undergo protracted development.^44,95^ Accelerated maturation could disrupt the delicate balance of their integration, predisposing an individual to affective dysregulation characteristic of anxiety and depression.^96^ This finding reframes our understanding of neurodevelopmental risk, suggesting that the pace of brain maturation is critical for long-term mental health. Maternal prenatal factors have been reported to significantly alter functional connectivity in infants,^1,36,97–99^ with maternal neglect and insensitivity associated with accelerated development of limbic regions.^100,101^ Although we did not detect any effects of MPD on brain growth trajectories, future research is needed to investigate other potential factors (maternal prenatal stress, maternal depression, substance exposure, etc.) that may additively contribute to accelerated maturation of brain connectivity.

We also found that elevated nodal and local efficiency at 1 year predicted poorer working memory at 8 years, which may reflect disrupted or delayed synaptic pruning. During the first year of life, visual and sensorimotor regions undergo an experience-dependent pruning process, eliminating redundant synaptic connections to streamline neural communication.^44,102^ Elevated nodal efficiency may indicate a disruption or delay in this essential pruning process; a network that retains too many local connections can become inefficient at supporting complex, integrative cognitive functions like working memory, which rely on coordinated communication between distant brain regions.^103^ This noisy, hyper-connected local circuitry may trap information processing within local modules, hindering flexible, distributed network activity required for storing and manipulating information online. Thus, our results point to potential neural mechanisms for how early disruptions in synaptic refinement can lay lead to later executive function deficits observed in various neurodevelopmental disorders. From a clinical perspective, these findings are profound: neuroimaging in the first year of life could potentially identify at-risk infants long before behavioral evidence of anxiety, depression, or working memory deficits become apparent, providing a window for early targeted interventions designed to support healthy neurodevelopmental trajectories. By understanding the specific nature of the atypical connectivity (e.g., accelerated maturation vs. disrupted pruning), interventions could be tailored to the individual’s specific risk profile.

Lastly, our brain-age modeling and functional brain age gap analysis further revealed disorder-specific developmental deviations. Our multifeatured deep learning model using six key functional connectivity features (functional connectivity maturation score, nodal global efficiency, nodal local efficiency, nodal strength, and functional gradient scores) consistently predicted chronological age of healthy cohort in the first two years of life. This suggests that functional connectivity most tightly reflects biological maturation during this critical period.^25^ The resulting brain age gap differentiated risk groups, with ADHD showing negative brain age gaps consistent with delayed maturation, and autism and MPD showing positive brain age gaps reflecting accelerated trajectories. The brain age gap metric also improved classification performance beyond functional connectivity features alone in distinguishing TD participants from neurodevelopmental risk groups (NDD and MPD). These patterns are consistent with prior evidence of heterogeneous developmental timing in neurodevelopmental disorders^104,105^ and suggest that functional brain age metrics may provide complementary, nonredundant information for early identification.

Several limitations warrant consideration. First, brain state differed across ages (natural sleep from 0-2, awake scanning from 4-10). From one perspective, this represents a limitation since this state change may potentially confound developmental trajectories. However, given that consistently scanning infants during awake state is practically extremely challenging,^106,107^ the current set of developmental trajectories with this brain state change likely represents the best and most practical reference curves for comparison and prediction purposes for future studies. Users of these reference curves should be mindful of this state change in interpreting related developmental changes. Second, rsfMRI scans were relatively short (3-5 minutes). While reliability declines with shorter acquisitions,^108^ prior work demonstrates that scans lasting between 3-12 minutes can still yield stable connectivity estimates,^109,110^ particularly in large samples.^111^ Indeed, our previous studies using 3-minute rsfMRI scans have detected robust functional connectivity effects.^25,27,112^ Third, despite efforts to harmonize acquisition protocols using ComBat,^113^ scanner differences may have introduced residual variance at 8 and 10 years of age. Fourth, the number of children later diagnosed with ADHD or autism was modest (n=36). Although previous studies indicate that group analyses with a minimum of 20 subjects can achieve acceptable reliability,^114^ future research focusing on ADHD and autism in larger clinical cohorts is necessary to validate our findings.

Future work should expand to examine genetic and environmental influences on functional connectivity development using approaches such as genome-wide association, transcriptomic analysis, and computational simulation.^6,115^ Denser longitudinal sampling, particularly in infancy and early childhood, will be important for capturing nonlinear dynamics of network maturation.^116^ Future studies should apply advanced deep learning methods in large, multisite cohorts to improve prediction of behavioral outcomes and neurodevelopmental disorders from functional connectome data, ultimately advancing early diagnosis and interventions through novel imaging biomarkers.

In conclusion, we established the first comprehensive normative trajectories of brain functional connectivity across the first decade of life to chart functional connectivity maturation, graph-theoretical organization, and functional gradient topology. Using a large longitudinal cohort and advanced connectomic modeling, we characterized dynamic shifts in network hierarchy and hub structure during this critical developmental window. We further identified altered trajectories in children later diagnosed with neurodevelopmental disorders and detected brain–behavior associations, underscoring the clinical relevance of early functional connectivity. Lastly, our brain age modeling revealed disorder-specific developmental deviations in brain age gap, and brain age gap significantly improved classification performance beyond functional connectivity features alone in distinguishing neurotypical participants from neurodevelopmental risk groups. This highlights functional brain age as a complementary, nonredundant marker for early detection of atypical development. Together, these findings provide a normative reference framework for future studies and offer a scalable approach for longitudinal connectome analysis to advance our understanding of typical and atypical brain development.

## Methods

### Participants

Infant participants were recruited as part of the University of North Carolina (UNC) at Chapel Hill Early Brain Development Study (EBDS), which focuses on early brain and behavioral development.^1,2^ Parental or legal guardian informed consent was obtained under protocols approved by the UNC Chapel Hill and Cedars-Sinai Medical Center Institutional Review Boards (UNC IRB #03-0989; Cedars-Sinai IRB Pro00042086). After data preprocessing (described below), we identified 633 children with at least one successful resting-state functional magnetic resonance imaging (rsfMRI) scan across the first 10 years of life. For twin pairs, one participant from each twin pair with the greatest number of good quality rsfMRI scans across all age groups was included. The final dataset included data collected at 3 weeks (n=372), 1 year (n=273), 2 years (n=220), 4 years (n=125), 6 years (n=175), 8 years (n=155), and 10 years of age (n=156). Participant demographics are summarized in Table S1. Of the 633 participants, 379 were typically developing (TD) controls. Inclusion criteria for the TD group included gestational age of ≥32 weeks at birth, no significant medical or neurological conditions, no history of maternal psychiatric diagnosis, and no neonatal illness requiring more than a 24-hour stay at a neonatal intensive care unit. Of the remaining 254 participants, children who received a diagnosis of ADHD or autism at 4 years of age were included in a neurodevelopmental disorder (NDD) subgroup (n=36), and children whose mothers had a psychiatric diagnosis (schizophrenia, schizoaffective, bipolar, major depression, psychosis, and mood disorders) were included in a maternal psychiatric diagnosis (MPD) subgroup (n=100). The remaining 118 participants (who did not meet criteria for TD, NDD, or MPD) were included in the longitudinal analyses to maximize representative and robust modeling of developmental trajectories.

RsfRMI data from 100 health adults (21-35 years old) were downloaded from the publicly available Human Connectome Project (HCP)^124^ to serve as an adult reference cohort for calculating maturation scores (described below). Eight participants were excluded after data preprocessing (described below), yielding a final cohort of 92 adults.

### Imaging Acquisition

Longitudinal rsfMRI data were collected at 3 weeks (neonates), 1 year, 2 years, 4 years, 6 years, 8 years, and 10 years of age. The distribution of available datasets for functional connectivity analyses is shown in Figure S3. Given the length of the parent study and scanner upgrades that occurred partway through, MRI data were collected across several scanners. Scanner effects were harmonized using ComBat^113^ to remove potential batch effects from different scanner types. All 0-6 year data and a portion of the 8-10 year data were collected on a Siemens 3T Allegra (circular polarization head coil) or Siemens 3T Tim Trio scanner (32-channel head coil). Most of the 8-10 year data were collected on a Siemens 3T Magnetom Prisma scanner (32-channel head coil). All neonate, 1-, and 2-year-old subjects were scanned during natural sleep, while all other age groups were scanned while awake. All 4-year-olds watched cartoons and 6-year-olds watched either cartoons or a fixation cross. For 8- and 10-year-olds, those scanned on the Siemens 3T Tim Trio watched movies, while those scanned on the Siemens 3T Magnetom Prisma were presented with a fixation cross.

Functional and structural images were acquired using the following sequences and parameters for each scanner type. Siemens 3T Allegra: functional images were acquired using a T2*-weighted echo planar imaging (EPI) sequence (TR=2000 ms, TE=32 ms, 33 slices, voxel size=4 mm^3^, 150 volumes); structural images were acquired using a T1-weighted 3D magnetization prepared rapid acquisition gradient-echo (MPRAGE) sequence (TR=1820 ms, TE=4.38 ms, inversion time=1100 ms, voxel size=1 mm^3^). Siemens Tim Trio: functional images were acquired using a T2*-weighted EPI sequence (TR=2000 ms, TE=32 ms, 32 slices, voxel size=4 mm^3^, 208 volumes); structural images were acquired using a T1-weighted 3D MPRAGE sequence (TR=1900 ms, TE=3.74 ms, inversion time=1100 ms, voxel size=1 mm^3^). A subset of images were collected on the Siemens 3T Tim Trio using an updated protocol: functional images were acquired using a T2*-weighted EPI sequence (TR=2630 ms, TE=32 ms, 40 slices, voxel size=3 mm^3^, 169 volumes); structural images were acquired using a T1-weighted 3D MPRAGE sequence (TR=2110 ms, TE=3.86 ms, inversion time=1100 ms, voxel size=0.8 mm^3^). Siemens 3T Magnetom Prisma: functional data were acquired using a T2*-weighted EPI sequence (AP and PA directions, along with single-band references; TR=800 ms, TE=37 ms, 72 slices, voxel size=2 mm^3^, 420 volumes); structural images were acquired using a T1-weighted 3D MPRAGE sequence (TR=2400 ms, TE=2.22 ms, inversion time=1000 ms, voxel size=0.8 mm^3^); spin-echo field maps (AP/PA) were also acquired for distortion correction.

According to the HCP documentation,^117,118^ structural images were acquired on a Siemens Skyra 3 T using a 3D T1-MPRAGE sequence: TR=2400 ms, TE=2.14 ms, inversion time=1000 ms, voxel size=0.7 mm^3^. Functional images were acquired using a gradient-echo EPI sequence: TR=720 ms, TE=33.1 ms, 72 slices, voxel size=2 mm^3^, 1200 volumes, multiband factor=8, and alternating runs of right-to-left (RL) and left-to-right (LR) phase encoding directions.

### Behavioral Measures at 8 and 10 Years of Age

Behavioral assessments were conducted at 8 and 10 years of age (Table S1) to examine cognitive and socioemotional outcomes including intelligence quotient (IQ), working memory, anxiety, and depression. Measures indexing cognitive development included 8- and 10-year abbreviated IQ (ABIQ) as assessed by the Stanford-Binet Intelligence Scales, 5th edition (SB-5)^119^ and working memory as assessed by the SB-5 and the Behavior Rating Inventory of Executive Function (BRIEF).^120^ Measures indexing socioemotional development included 10-year anxiety and depression assessed using Behavior Assessment System for Children, 2nd edition (BASC-2).^121^

The SB-5 is a set of individually administered tasks conducted in a structured environment. These scales are designed to measure intelligence across the lifespan, with a specific focus on five key domains (Fluid Reasoning, Knowledge, Quantitative Reasoning, Visual-Spatial Processing, and Working Memory). Here, we focused on the ABIQ and working memory scores as the primary outcomes of interest. The ABIQ score provides a measure of general cognitive ability and is derived from performance on two subtests (a nonverbal test assessing object or sequence/pattern recognition, a verbal test assessing vocabulary), with higher scores indicating better cognitive capabilities. The working memory score provides a measure of underlying working memory skills as assessed across a verbal and nonverbal working memory task, with higher scores indicating better working memory capability.

The BRIEF, a questionnaire completed by parents and teachers to assess executive function behaviors in children and adolescents, is comprised of two indexes: Behavioral Regulation (Inhibit, Shift, and Emotional Control subscales) and Metacognition (Initiate, Working Memory, Plan/Organize, Organization of Materials, and Monitor subscales). We focused on the working memory subscale, which assesses the application of working memory skills in daily situations. Higher BRIEF working memory scores indicate a greater degree of executive dysfunction (i.e., worse working memory capability).

The BASC-2 parent-report form (child version) includes nine clinical scales (Hyperactivity, Aggression, Conduct Problems, Anxiety, Depression, Somatization, Atypicality, Withdrawal, and Attention Problems). For each scale, item scores are totaled and converted into T-scores standardized by age and sex. We focused on the anxiety and depression scores. The anxiety scale assesses worry or fear related to real or imagined problems, and the depression scale assesses sadness, loneliness, hopelessness, and a lack of interest in previously enjoyed activities. Higher BASC-2 anxiety and depression scores indicate higher symptom levels. A T-score ≥ 60 indicates that a child is “at risk,” and a score ≥ 70 is classified as “clinically significant.” A high score on the anxiety or depression scale alone is generally insufficient for clinical diagnosis, but higher clinical scales reported on the BASC-2 have been shown to be associated with other neurodevelopmental disorders, including autism.^122^

### fMRI Data Preprocessing

Functional imaging data were preprocessed using FMRIB’s Software Library (FSL),^123^ Advanced Normalization Tools (ANTs),^124^ and Analysis of Functional Neuroimages (AFNI), ^125^ using a preprocessing pipeline developed in prior work.^126^ Preprocessing steps included rigid-body motion correction, registration, bandpass filtering (0.01–0.10 Hz), motion scrubbing, nuisance signal regression, and global signal regression. For data collected on the Siemens 3T Magnetom Prisma, AP/PA concatenation was performed. For motion correction, the first frame of the functional image (or the single-band reference image for Siemens 3T Magnetom Prisma data) was used as the target image, and framewise displacement (FD) was calculated from six motion parameters (displacements and rotations). The nuisance signal regression model included 32 parameters: 8 regressors (including white matter and cerebrospinal fluid signals) and 24 motion-related parameters (six motion correction parameters, their derivatives, and their quadratic terms).^127^ Data scrubbing was performed as an additional motion correction step by removing volumes with FD>0.3 mm; in cases where there were fewer than three remaining volumes between two removed/scrubbed volumes, these were also removed.^128^ Participants with fewer than 3 minutes of data remaining after scrubbing were excluded from analysis (n=17).^139^ Functional datasets were aligned to the age-specific template^129^ through per-volume transformations, including rigid-body motion correction within functional space, nonlinear functional-to-anatomical registration, and nonlinear anatomical-to-standard registration. Nonlinear distortion correction was performed for Siemens 3T Magnetom Prisma data. ANTs^124^ was used for the nonlinear registrations, with an age-specific template^129^ serving as the intermediate target space. Specifically, we first computed transformations from the native functional space to the native structural T1 space, followed by transformations from the native T1 space to the template space. Visual inspections ensured successful registration for each subject. Following transformation to the standard space, data were spatially smoothed with a Gaussian kernel of 6-mm full width at half maximum (FWHM). A 2-year template^129^ was used as the final target for spatial registration, ensuring alignment of rsfMRI data across all ages to a consistent template space for subsequent analyses. Finally, global signal regression was applied to remove the mean gray matter signal from the data.

Adult HCP data were preprocessed following this protocol except for the following: 1) RL/LR session concatenation was performed for rsfMRI data; 2) the MNI-152 adult template^130^ served as an intermediate target space prior to registration to the 2-year template.

### Functional Connectivity Analysis

The overall schematic of our methodological approach is shown in Figure S4. After preprocessing, we generated voxelwise 15,450*15,450 functional connectivity matrices using the 2-year template brain mask (4 mm^3^ resolution).^129^ Specifically, across all 15,450 voxels, pairwise Pearson correlations were calculated between the timeseries of each voxel and that of every other voxel. These correlation values were Fisher-*Z* transformed, resulting in a voxelwise functional connectivity matrix for each subject. A group-level voxelwise functional connectivity matrix was calculated across all HCP subjects in the 2-year template space (i.e., mean across 92 subjects); this served as an adult reference for the maturation scores (described below).

Using these functional connectivity matrices, we calculated the following functional connectivity parameters for each voxel (details described below): maturation scores, graph theory measures (nodal strength, nodal global efficiency, and nodal local efficiency), and functional gradient scores.^38^ For all functional connectivity parameters, voxelwise Combat^113^ followed by linear regression (functional connectivity measure ~ 1 + mean FD + scan length) was conducted to harmonize across scan-related effects (scanner, mean FD, scan length). Briefly, Siemens 3T Trio-Tim scanner was set as the base category for Combat since all age groups included subjects that were scanned on this scanner. Other continuous variables (mean FD and scan length) were mean centered. Combat and regression were then conducted within each age group to estimate scan-related effects. The 8- and 10-year data were combined for harmonization since these age groups shared similar functional connectivity heatmaps and only six 10-year-old participants were scanned on the base scanner (Siemens 3T Trio-Tim scanner). After regression, only the sum of the intercept and the residual for each voxel remained to rule out the scan-related effects. The resulting individual-level heatmaps were averaged within the TD group to obtain normative developmental heatmaps for each functional connectivity measure across 0-10 years of age.

Next, network-level functional connectivity features for each measure were derived from individual-level heatmaps using a 7-network parcellation mask^131^ (visual, sensorimotor, dorsal attention, ventral attention/salience, limbic, frontoparietal, and default mode networks) as well as a subcortical network (including hippocampus, parahippocampal gyrus, amygdala, caudate, putamen, pallidum, and thalamus). Functional parcellation region of interest (ROI)-level functional connectivity features were derived from a 2-year functional parcellation template.^132^

### Functional Connectivity Parameter Calculation

#### Functional Connectivity Maturation Score

A functional connectivity maturation score (Mscore) was calculated to assess the maturation of functional connectivity from 0 to 10 years of age. The mean adult functional connectivity matrix was used as a reference for the maturation score calculation. A filtered reference matrix was generated by retaining only the top 10% of positive functional connectivity values for each voxel (i.e., for each column) in the mean adult functional connectivity matrix. For each child, a filtered functional connectivity matrix was generated by retaining only the 10% of positive functional connectivity values for each voxel (i.e., for each column in the functional connectivity matrix). A maturation score was then calculated for each voxel; this was defined as the cosine similarity of the filtered functional connectivity matrix between each participant and the adult reference.

#### Graph Theory Measures

Graph theory measures, including nodal strength, nodal global efficiency, and nodal local efficiency,^133^ were derived for each node from the weighted functional connectivity matrices. To avoid shared signals between adjacent voxels and to facilitate subsequent computations, functional connectivity matrices were resampled to 8 mm^3^. Individual nodes were defined as regions pooled together across eight adjacent voxels from the original 4 mm^3^ resolution space. These functional connectivity matrices were filtered to retain only the top 10% positive functional connectivity values to reduce noise and maintain consistent density across age groups.^134^ Nodal strength, nodal global efficiency, and nodal local efficiency were derived from the filtered functional connectivity matrices using established definitions as described in the Brain Connectivity Toolbox.^133^ We implemented these calculations using a customized R script based on the brainGraph and igraph packages^135,136^ to boost computational efficiency. Nodal strength is a measure of overall connectivity of a given node, defined as the sum of the weights of all edges connected to a node. Nodal global efficiency is a measure of how efficiently a node can communicate with all other nodes, defined as the average inverse shortest path length between a given node and all other nodes. Nodal local efficiency is a measure of information transfer efficiency within the immediate neighborhood of a node and measures a network’s resilience to the removal of individual nodes (i.e., communication efficiency among a node’s immediate neighbors using only the connections between those neighbors), defined as the average of the global efficiency of subgraphs formed by the neighbors of each node.^133^

#### Functional Gradients

Brain functional gradients provide a low-dimensional representation of the brain’s complex functional connectivity patterns.^38,39,137^ We computed functional gradients from voxelwise 15,450*15,450 functional connectivity matrices using the BrainSpace toolbox^138^ following established protocols.^38,68,69,138^ A filtered functional connectivity matrix was first generated by retaining only the top 10% of positive functional connectivity values of each row (i.e., each voxel). For each participant, an affinity matrix was then determined by calculating the cosine similarity across all rows (i.e., all voxels) of the filtered functional connectivity matrix. Ten functional connectivity gradients were computed for each subject by applying diffusion map embedding^139^ with α=0.5 to the affinity matrix. Individual gradients across all age groups were aligned to adult reference gradients using iterative Procrustes alignment^138^ (including only rotations and reflections to retain the original scale of the gradients). We focused on the first two functional gradients: the sensorimotor-to-visual gradient (Gradient1) and the primary-to-transmodal gradient (Gradient2).^38,68,69^ Gradient plots were obtained by graphing voxelwise Gradient1 and Gradient2 values to illustrate the topology and proximity of functional gradients, with each voxel color-coded based on its network membership. It is worth noting that the sign of the gradient score is arbitrary; axes were flipped in the figures for ease of comparison with extant literature.^68,69,73^

Other functional gradient measures were also assessed, including gradient range as well as whole-brain and network-level functional gradient dispersion. Gradient range was calculated as the absolute value of the difference between the minimum and maximum across all voxels for each gradient (Gradient1 and Gradient2). This measures the extent to which functional connectivity is differentiated along a specific gradient axis. Dispersion was calculated as the mean Euclidean distance between the gradient coordinates of all voxels and the centroid.^41^ This metric is invariant to Procrustes alignment without scaling, as the internal structure and topology remain unchanged. We assessed dispersion of Gradient1 and Gradient2 at both the whole-brain (using all voxels across the entire brain) and network levels (using only voxels within each network).

### Functional Connectivity Longitudinal Analysis

To model the longitudinal trajectories of network- and ROI-level functional connectivity features, we used a multivariate sparse functional principal components analysis (mSFPCA) method.^40^ This approach uses cubic spline basis functions (with a single knot positioned at the one-third quantile of the time distribution) to fit the mean and principal component functions. Nonlinear longitudinal trajectories are characterized as a smooth mean function combined with a weighted sum of the primary smooth modes of variation around the mean. We retained four functional principal components (FPCs), and the FPC scores (i.e., weights) were jointly modeled using a constrained covariance matrix estimated through Cholesky decomposition. This mSFPCA framework was applied to all subjects to extract the overall mean, FPCs, and individual-level FPC scores, which were then used to reconstruct individual-level trajectories. Group-level developmental trajectories were calculated for each group (TD, NDD, MPD).

Statistical group comparisons were conducted via linear regression on the union of the TD, NDD, and MPD groups both longitudinally (using the FPC scores) and cross-sectionally (using functional connectivity features at each age point). Finally, the relationships between functional connectivity features at each age point and 10-year behavioral outcomes were assessed using partial correlation and a permutation test.

### Between-Group Comparisons

Group comparisons were conducted both cross-sectionally and longitudinally. In the cross-sectional analysis, we focused on network- and ROI-level functional connectivity measures for each age group. Linear regression (functional connectivity parameter ~ 1 + gestational age at birth + gestational age at scan + birth weight + sex + maternal education + NDD + MPD) was conducted across all TD, NDD, and MPD participants. False discovery rate (FDR) was used to correct for multiple comparisons. In the longitudinal analysis, we focused on the first FPC score since this captured most of the variance. Linear regression (FPC score of the FPC parameter ~ 1 + gestational age at birth + birth weight + sex + maternal education + NDD + MPD) was conducted across all TD, NDD, and MPD participants.

### Brain–Behavior Prediction

Brain–behavior relationships were assessed using a canonical correlation analysis (CCA) to see whether ROI-level functional connectivity features (*z*-scored by normative trajectories) at 0, 1, and 2 years predicted behavioral outcomes at 8 and 10 years of age (SB-5 ABIQ, SB-5 working memory, BRIEF working memory, BASC-2 anxiety, BASC-2 depression). Covariates of no interest included gestational age at birth, gestational age at scan, birth weight, sex, maternal education and gestational age at the 8/10-year behavioral assessment. We trained a principal components analysis (PCA)-CCA model on TD participants, then tested the model in a pooled risk group including NDD and MPD participants. Permutation tests were conducted to determine significance of the CCA predictions in the test set. To establish a null distribution, behavioral scores across the participants were randomly permuted 1000 times to train 1000 PCA-CCA null models and generate 1000 test correlation values between the brain functional connectivity latent variable and the permuted behavioral latent variable. A permuted *p*-value (*p_perm_*) was then calculated as the number of permutations in the null distribution with a more extreme correlation value (i.e., greater in absolute value) compared with the true correlation value computed with the correctly labeled data divided by the total number of permutations. FDR correction was additionally performed to correct for multiple tests. The loadings of each sub-feature for each modality (brain/behavior) were also calculated as the correlation of the latent variable (linear combination of all sub-features) and each of its corresponding sub-features.

### Predicting Brain Age with a Deep Learning Model

A deep-learning based regression model was trained to estimate age from the functional connectivity measures by extracting age-sensitive features for clinical analysis.^140^ The TD group was split into training and testing subsets that included datasets (i.e., scans) across all age groups (9:1 split; train on n=1257 scans, test on n=126 scans). For each participant, we used six whole-brain heatmap inputs: maturation score, nodal strength, nodal global efficiency, nodal local efficiency, sensorimotor-to-visual gradient (Gradient1) scores, and primary-to-transmodal gradient (Gradient2) scores. Each map was stacked, axial-sliced (45 2D slices; 45 x 54 pixels), and min–max rescaled to [-1,1]. We trained six single-input models (one per heatmap) and one concatenated model using all six heatmaps as stacked channels. The model architecture included a 2D ResNet^141^ backbone with four residual stages (channels 32/64/128/256; kernel 3x3; strides 2/2/1/1), followed by global average pooling,^142^ two fully connected layers, and a final softmax over 1024 age bins.^140^ The softmax output p is interpreted as a discrete age distribution over 0–4096 gestational days (uniform binning; 0-10 years of age). Training was supervised using Kullback–Leibler divergence^143^ between the output p and a Gaussian target (mean=true gestational age, standard deviation=90 days for soft labels) as loss function. Main training parameters included an Adam (with learning rate 4e-4) optimizer,^144^ cosine annealing scheduler,^145^ batch size 512 (slices), and 200 epochs. Slice-level predictions were aggregated during inference as follows: for the 20 central slices (chosen to retain brain coverage and suppress edging slice effects), we obtained the subject-level age by sorting the predictions of the 20 slices and taking the mean value of the middle 10 slices as global age prediction. Performance was evaluated by mean absolute error (MAE) on the held-out test set.^146^

We next conducted a brain age gap (BAG) analysis to assess how different risk factors may impact predictions of brain age.^92,147^ Since accuracy of the brain age prediction model previously trained on the six functional connectivity heatmaps was highest between birth and 2 years of age, we conducted the BAG analysis at 3 weeks (indicated as 0 year), 1 year, and 2 years of age and calculated predicted brain age of participants with ADHD, autism, or MPD. BAG was defined as $\mathrm{BAG}=\hat{a}-a$, where $\hat{a}$ stands for predicted age and *a* is chronological (gestational) age. To test case-control differences in BAG, we applied two-sided Mann–Whitney U tests^148^ comparing ADHD (n=31 with rsfMRI data at 0, 1, and 2 years of age), autism (n=23), and MPD (n=141) subgroups to TD children (n = 65) at 0, 1, and 2 years of age.

Lastly, we trained binary classifiers to distinguish between membership in the TD group or pooled neurodevelopmental risk group (NDD and MPD). The evaluation pool included the brain age test set (n=65 TD children with rsfMRI data at 0, 1, and 2 years of age) plus all participants with ADHD, autism, or MPD (n=174). Functional connectivity features were derived from six functional connectivity features (maturation score, nodal strength, nodal global efficiency, nodal global efficiency, sensorimotor-to-visual gradient [Gradient1] score, and primary-to-transmodal gradient [Gradient2] score) across eight canonical brain networks (visual, sensorimotor, dorsal attention, ventral attention/salience, limbic, frontoparietal, default mode, subcortical networks), yielding 48 functional connectivity features per rsfMRI dataset (i.e., per scan). To mitigate overfitting due to the high feature-to-sample ratio, we applied principal component analysis (PCA) to the 48 functional connectivity features and retained 5 components. BAG (i.e., difference between the predicted age and chronological age) was appended as a sixth feature. The PCA transformation was fit on training data only and applied to the test data to prevent data leakage. We employed a subject-level stratified 10-fold cross-validation scheme to ensure that all datasets from one subject (across age groups) appeared in the same fold and that class proportions were preserved. We evaluated 12 linear classifiers including logistic regression (L1, L2, elastic net at multiple regularization strengths), ridge classification, linear support vector machine (SVM), and stochastic gradient descent variants, all with balanced class weights.^149^ We repeated the pipeline across 10 random seeds and selected the model that achieved the highest mean cross-validated accuracy for each seed. To isolate the contribution of BAG to the prediction, we compared three feature conditions on the same 200 seeds: 1) FC+BAG model, including the five functional connectivity principal components and BAG, 2) FC-only model, including only the five functional connectivity principal components, and 3) FC+Random model, including the five functional connectivity principal components and a random Gaussian feature matched to the mean and standard deviation of BAG. This random feature control served to rule out the possibility that model improvement was due to merely adding an extra feature dimension. We ran paired *t*-tests and Wilcoxon signed-rank tests across all matched seeds. Finally, to quantify feature contributions, we computed Shapley Additive explanation (SHAP) values using LinearExplainer for linear models across all 10 folds for each of the top 10 seeds. Mean absolute SHAP values were averaged across folds and seeds to produce a final feature importance ranking.

## Supplementary Material

This is a list of supplementary files associated with this preprint. Click to download.

• NatHumBehavSupplement20260422.pdf

## Figures and Tables

**Table 1.** Performance metrics across classification models.

Model	Accuracy	F1 Score	AU-ROC
FC+BAG	0.61 ± 0.02	0.53 ± 0.03	0.58 ± 0.02
FC-only	0.57 ± 0.02	0.49 ± 0.02	0.52 ± 0.04
FC+Random	0.56 ± 0.03	0.48 ± 0.04	0.51 ± 0.05
